# Air-Adapted *Methanosarcina acetivorans* Shows High Methane Production and Develops Resistance against Oxygen Stress

**DOI:** 10.1371/journal.pone.0117331

**Published:** 2015-02-23

**Authors:** Ricardo Jasso-Chávez, M. Geovanni Santiago-Martínez, Elizabeth Lira-Silva, Erika Pineda, Armando Zepeda-Rodríguez, Javier Belmont-Díaz, Rusely Encalada, Emma Saavedra, Rafael Moreno-Sánchez

**Affiliations:** 1 Departamento de Bioquímica, Instituto Nacional de Cardiología Ignacio Chávez, Mexico City, Mexico; 2 Facultad de Medicina, UNAM, Mexico City, Mexico; Louisiana State University Health Sciences Center, UNITED STATES

## Abstract

*Methanosarcina acetivorans*, considered a strict anaerobic archaeon, was cultured in the presence of 0.4–1% O_2_ (atmospheric) for at least 6 months to generate air-adapted cells; further, the biochemical mechanisms developed to deal with O_2_ were characterized. Methane production and protein content, as indicators of cell growth, did not change in air-adapted cells respect to cells cultured under anoxia (control cells). In contrast, growth and methane production significantly decreased in control cells exposed for the first time to O_2_. Production of reactive oxygen species was 50 times lower in air-adapted cells *versus* control cells, suggesting enhanced anti-oxidant mechanisms that attenuated the O_2_ toxicity. In this regard, (i) the transcripts and activities of superoxide dismutase, catalase and peroxidase significantly increased; and (ii) the thiol-molecules (cysteine + coenzyme M-SH + sulfide) and polyphosphate contents were respectively 2 and 5 times higher in air-adapted cells *versus* anaerobic-control cells. Long-term cultures (18 days) of air-adapted cells exposed to 2% O_2_ exhibited the ability to form biofilms. These data indicate that *M. acetivorans* develops multiple mechanisms to contend with O_2_ and the associated oxidative stress, as also suggested by genome analyses for some methanogens.

## Introduction

The reactive oxygen species (ROS) are toxic for most cells because they induce (i) oxidation of polysaccharides and polyunsaturated fatty acids, as well as amino acid residues, particularly of sulfhydryl groups in proteins; (ii) loss of metals in metalloproteins; and (iii) DNA mutations, among many others [1]. Aerobic microorganisms have developed multiple strategies to handle ROS stress including: (i) enzymes that scavenge ROS such as superoxide dismutase (SOD), catalase (CAT) and peroxidases (PXs); (ii) protein repair mechanisms such as the thioredoxin system; (iii) DNA damage repair enzymes such as RecA; and (iv) anti-oxidant metabolites such as glutathione, α-tocopherol, carotenes, ascorbate, and trypanothione, which are able to directly inactive ROS [1–4].

The organisms belonging to the *Archaea* domain generally live under extreme conditions [5]. Indeed, many live under complete anaerobic conditions; therefore, it has been frequently assumed that most anaerobic archaea do not interact with O_2_ and therefore they lack mechanisms able to cope with oxidative stress.

Methanogens, the main *Archaea* group, grow in anoxic environments such as the rumen, sewage digesters, landfills, freshwater sediments of lakes and rivers, rice paddies, hydrothermal vents and coastal marine sediments [6]. Therefore, most of the methanogens are cultivated in the presence of high Na_2_S (1–3 mM) to yield an anoxic and reducing medium (-300 mV).

Biochemical and genetic (genome and transcriptome) analyses have suggested that methanogens have the ability to develop mechanisms to cope with oxidative stress [7]. Methanogens such as *Methanosarcina spp* and *Methanocella spp* have been isolated from soil crusts of arid regions where aerobic conditions are predominant [8]. In these places, methane production by these methanogens is detected, but methanogenic rates are much lower when O_2_ is present. Increased transcription of the peroxide-detoxifying *kat* gene (catalase) was found in these methanogens, but the enzyme activity was not determined [9].

*Methanobrevibacter arboriphilicus* SA, *Methanobacterium fomicicum* and *Methanosarcina mazei* TMA isolated from paddy soils are able to deal with periods of aeration and water stress for up to 30 days [10]. Analyses of the genomes of these methanogens show the presence of genes encoding antioxidant enzymes, which may be the main reason of the different abilities to resist aerobic conditions, rather than differences in the habitats that may act as shelters for methanogens during the long-term stress period.

In *Methanobrevibacter cuticularis* and *Methanobrevibacter curvatus* isolated from microaerofilic regions of the hindgut of termites, CAT and SOD activities are detected [11]; however, these organisms immediately cease growth and methane production when the cultures are initiated in the presence of 0.16–1.6% O_2_ in the head space [12]. In *Methanosarcina barkeri*, pulses of H_2_O_2_, but not of O_2_, induce the activity of both CAT and Fe-dependent SOD [13–15]. *Methanosarcina mazei* contains a methanoferrodoxin with superoxide reductase activity which contributes to the protection of cells from ROS formed by flavoproteins during periodic exposure to oxygen in natural environments [16]. The marine archaeon *Methanosarcina acetivorans* WWM73 strain can tolerate high H_2_O_2_ concentrations without a complete loss of viability [17]. Also, a functional thioredoxin reductase system has been reported for this methanogen [18].

*Methanosarcina spp* and *Methanosaeta spp* are the only methanogens able to consume acetate for methane production [19], which may account for 75% of the biological methane on earth. Despite this crucial role in the carbon cycle, knowledge regarding the mechanisms present in *Methanosarcina spp* to contend against oxidative stress is still incomplete. To assess the mechanisms of resistance against oxidative stress in methanogens, *M*. *acetivorans* was adapted to grow in the presence of permanent low O_2_ (0.4–1% O_2 atmospheric_). These air adapted cells showed increased transcripts of *sod*, *kat* and NADH-dependent peroxidase genes and activities of SOD, CAT and NAD(P)H-, cytochrome *c*- and CoM-SH-dependent peroxidases (PXs). An increase in the contents of thiol molecules and polyP was also observed. Moreover, long exposures (up to 18 days) to higher O_2_ concentrations led to formation of biofilms constituted by DNA, CHOs and proteins. The physiological relevance of these mechanisms in methanogens to cope with O_2_ in a marine environment is discussed.

## Results

### 2.1 Analyses of methanogenic genomes

A survey of genes coding for enzymes putatively involved in oxidative stress in genomes from 27 different genera belonging to the 5 orders of methanogens, available in the KEGG data base, showed that *M*. *acetivorans* is among the methanogens with the largest number of genes coding for anti-oxidant proteins (Table 1). SODs, catalases and PXs play an essential role in defending the cell against oxidative stress and are distributed in almost all aerobic and facultative anaerobic organisms. In this regard, it has been proposed that the presence or absence of one or both of the SOD and CAT activities determines whether an anaerobe is aerotolerant [20]. The results shown below indicated that in *M*. *acetivorans* one of the mechanisms underlying the anti-oxidant response was the expression of antioxidant enzymes that are not present in all methanogens.

**Table 1:** Genes annotated coding for proteins involved in oxidative stress protection in methanogens.

*Genera*	*kat*	*sod*	*px*	*rbr*	dfx	*rbx*	*fprA*	*trx*	*trxr*	*prx*	*grx*	*cyd*
*Methanocaldococcus*	N	N	N	Y	Y	Y	Y	Y	Y	Y	N	N
*Methanotorris*	N	N	N	Y	Y	Y	N	Y	Y	Y	N	N
Methanococcus*	N	N	Y	Y	Y	Y	N	Y	Y	Y	Y	N
*Methanothermococcus*	N	N	N	Y	Y	Y	N	Y	Y	Y	Y	N
Methanosarcina*	Y	Y	Y	Y	Y	Y	Y	Y	Y	Y	Y	Y
*Methanococcoides*	Y	N	Y	Y	Y	Y	N	Y	Y	Y	Y	N
*Methanohalophilus*	Y	N	Y	Y	Y	N	N	Y	Y	Y	Y	N
Methanohalobium*	N	N	Y	Y	Y	Y	N	Y	Y	Y	Y	N
*Methanosalsum*	Y	Y	Y	Y	Y	N	N	Y	Y	Y	Y	N
Methanolobus*	Y	Y	Y	Y	N	N	N	Y	Y	Y	Y	N
*Methanomethylovorans*	Y	Y	Y	Y	Y	N	N	Y	Y	Y	Y	N
Methanosaeta*	Y	Y	Y	Y	Y	Y	N	Y	Y	Y	Y	N
*Methanospirillum*	Y	Y	Y	Y	Y	Y	N	Y	Y	Y	Y	N
*Methanocorpusculum*	Y	N	N	N	N	N	N	Y	N	Y	N	N
Methanoculleus*	Y	Y	Y	Y	Y	Y	N	Y	Y	Y	Y	N
Methanoplanus*	N	N	Y	Y	Y	Y	N	Y	Y	Y	Y	N
*Methanoregula*	Y	Y	Y	Y	Y	Y	N	Y	Y	Y	Y	N
*Methanosphaerula*	Y	N	Y	Y	Y	Y	N	Y	N	Y	Y	N
*Methanocella*	Y	Y	N	Y	Y	Y	Y	Y	Y	Y	Y	N
*Methanomassiliicoccus*	Y	N	N	Y	Y	Y	N	Y	Y	Y	N	N
*Methanothermobacter*	N	Y	N	Y	N	Y	Y	Y	Y	Y	Y	N
*Methanosphaera*	N	N	N	Y	Y	Y	Y	Y	Y	N	N	N
*Methanobrevibacter*	Y	Y	Y	Y	Y	Y	Y	Y	Y	N	N	N
Methanobacterium*	Y	Y	Y	Y	Y	Y	Y	Y	Y	Y	Y	N
*Methanothermus*	N	N	N	Y	Y	N	N	Y	Y	Y	N	N
*Methanopyrus*	N	N	N	Y	N	N	N	Y	Y	Y	N	N
*Methanomethylophilus*	Y	N	Y	Y	N	Y	N	Y	Y	Y	N	N

Genes present (Y) or absent (N) in the different genera of methanogens. *kat*: catalase; *sod*: superoxide dismutase; *px*: peroxidase; *rbr*: rubrerythrin; *dfx*: desulfoferrodoxine (superoxide reductase activity; SOR); *rbx*: rubredoxin; *fprA*: type A flavoprotein (F_420_H_2_ oxidase activity); *trx*: thiorredoxin; *trxr*: thioredoxin reductase; *grx*: glutaredoxin; *prx*: peroxiredoxin; *cyd*: cytochrome *d* oxidase. Asterisk denotes genera with marine species.

### 2.2 Effect of oxygen on growth and methane production

To determine the mechanisms of defense against oxidative stress in *M*. *acetivorans* as well as the effect of long-term exposure to O_2_, air-adapted cells were generated by periodic injections of air into the anoxic cell cultures for at least 6 months. To assess whether the resistance mechanisms against oxidative stress vary with the carbon source [7]; in the present work cells were also cultured with acetate or methanol for comparison.

**Fig 1:**
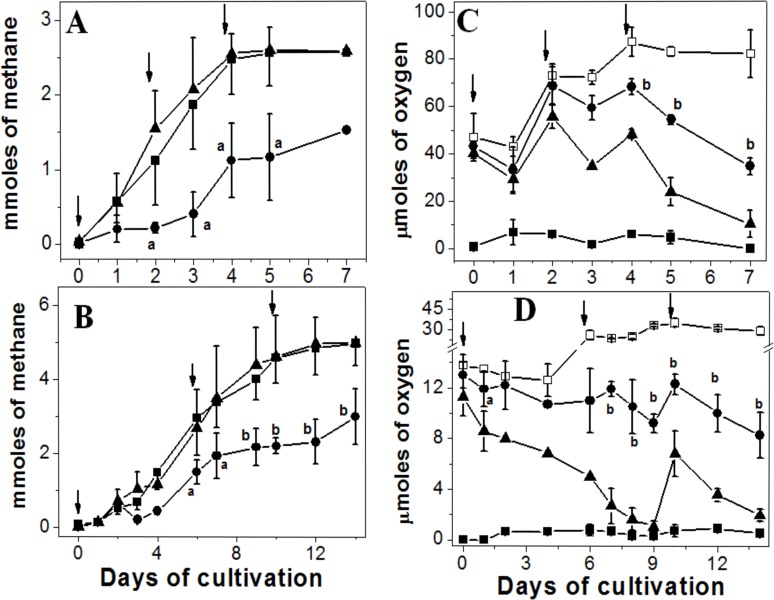
Methane synthesis and oxygen consumption in *M*. *acetivorans*. Cells were cultured in methanol (A, C) or acetate (B, D) and the contents of methane (A, B) and O_2_ (C, D) in the head space at the indicated times were determined. Control anaerobic cells (■), control anaerobic cells *plus* air pulses (●) and air-adapted cells (▲). The O_2_ concentration in the head space of culture bottles without cells was also determined (□). The increase in the content of O_2_ was due to each new air injection which was indicated by arrows. Values are the mean ± SD of at least 4 different independent cell batches. ^a^ P<0.05; ^b^ P <0.01 *vs* air-adapted cells.

*M*. *acetivorans* was able to contend with air exposure as judged by the similar methane synthesis rate in air-adapted cells *versus* control anaerobic cells. In contrast, anaerobic control cells subjected for the first time to air injections showed 40% less methane production in both, methanol and acetate cultures (Fig. 1A and 1B), and decreased protein contents: 4.1 ± 0.1 and 3.4 ± 0.3 mg/culture for methanol- and acetate-cell cultures, respectively, which meant a decrease of 35–40% respect to control cultures non-exposed to O_2_ (Table 2).

**Table 2:** Cellular responses to O_2_ in stationary growth phase in anaerobic control and air-adapted cells.

	Methanol anaerobic-control cells	Methanol air-adapted cells	Acetate anaerobic-control cells	Acetate air-adapted cells
Protein content (mg/ 50 mL culture)	6.9 ± 0.6 (5)	6.9 ± 0.4 (5)	5.2 ± 0.7 (5)	5 ± 1.3 (5)
Methane production (mmol / 50 mL culture)	2.5 ± 0.4 (5)	2.6 ± 0.3 (5)	5 ± 0.2 (5)	5 ± 0.1 (5)
Cysteine (nmol/mg total protein)	50 ± 10 (4)	80 ± 20 (4)	2.7 ± 0.6 (4)	2 (2)
Co-MSH (nmol/mg total protein)	5 ± 3 (4)	10 ± 2 (4)	15 ± 5 (4)	13 ± 3 (4)
Sulfide (nmol/mg total protein)	17 ± 10 (4)	42 ± 8* (4)	14 ± 3 (4)	48 ± 8* (4)
Inorganic phosphate (μmol/mg total protein)	0.88 ± 0.12 (6)	0.5 ± 0.11 (6)	1.3 ± 0.3 (6)	1.27 ± 0.28 (4)
PolyP (μmol/mg total protein)	0.75 ± 0.24 (6)	1.7 ± 0.4** (6)	1.62 ± 0.25 (4)	8.6 ± 2** (4)

Values of PolyP in nmol (mg protein)^-1^ for methanol cultures were: 62 ± 20 and 138 ± 35 for control and air adapted cells, respectively; for acetate cultures: 345 ± 53 and 2,190 ± 528 for control and air adapted cells, respectively. * P<0.05 *vs* anaerobic control cells. ** P<0.01 *vs* anaerobic control cells. Values shown are the mean ± SD; number of independent experiments is shown in parenthesis.

Most of the injected O_2_ was detected in the head space of the culture bottles (91 and 72% of total O_2_ for methanol- and acetate-cultures, respectively) at the beginning of the culture. Control anaerobic cultures showed negligible contamination by atmospheric O_2_ at the moment of the cell inoculum injection: 0.3–0.6 μmol O_2_ in 50 ml head space in methanol and acetate cell cultures (Fig. 1C and 1D). After the three air injections, the concentration of dissolved O_2_ in the medium at the end of the growth curve was 3.4 ± 0.5 and 5.3 ± 0.5 μM (mean ± SD, n = 3) for methanol- and acetate- air-adapted cell cultures, respectively. Control anaerobic cultures showed negligible levels of dissolved O_2_ (< 1.5 μM O_2_).

Anaerobic control and air-adapted cells showed ability to consume the added O_2_ (Fig. 1C and 1D). The rates of O_2_ consumption determined in methanol grown cells after the third air pulse were 14.9 ± 2 and 24 ± 3 μmol O_2_ day^-1^ (days 4 to 5) for anaerobic control and air-adapted cells, respectively; this is 63% faster for air-adapted cells (mean ± SD, n = 4). In turn, the rates of O_2_ consumption in acetate grown cells (days 10 to 12) were 1.07 ± 0.11 and 1.63 ± 0.3 μmol O_2_ day^-1^, for anaerobic control and air-adapted cells, respectively (n = 4); this means 52% faster for the latter cells. The protein content at the end of the growth curve for anaerobic control and air-adapted cells was essentially identical in both, methanol and acetate cultures (Table 2).

**Fig 2:**
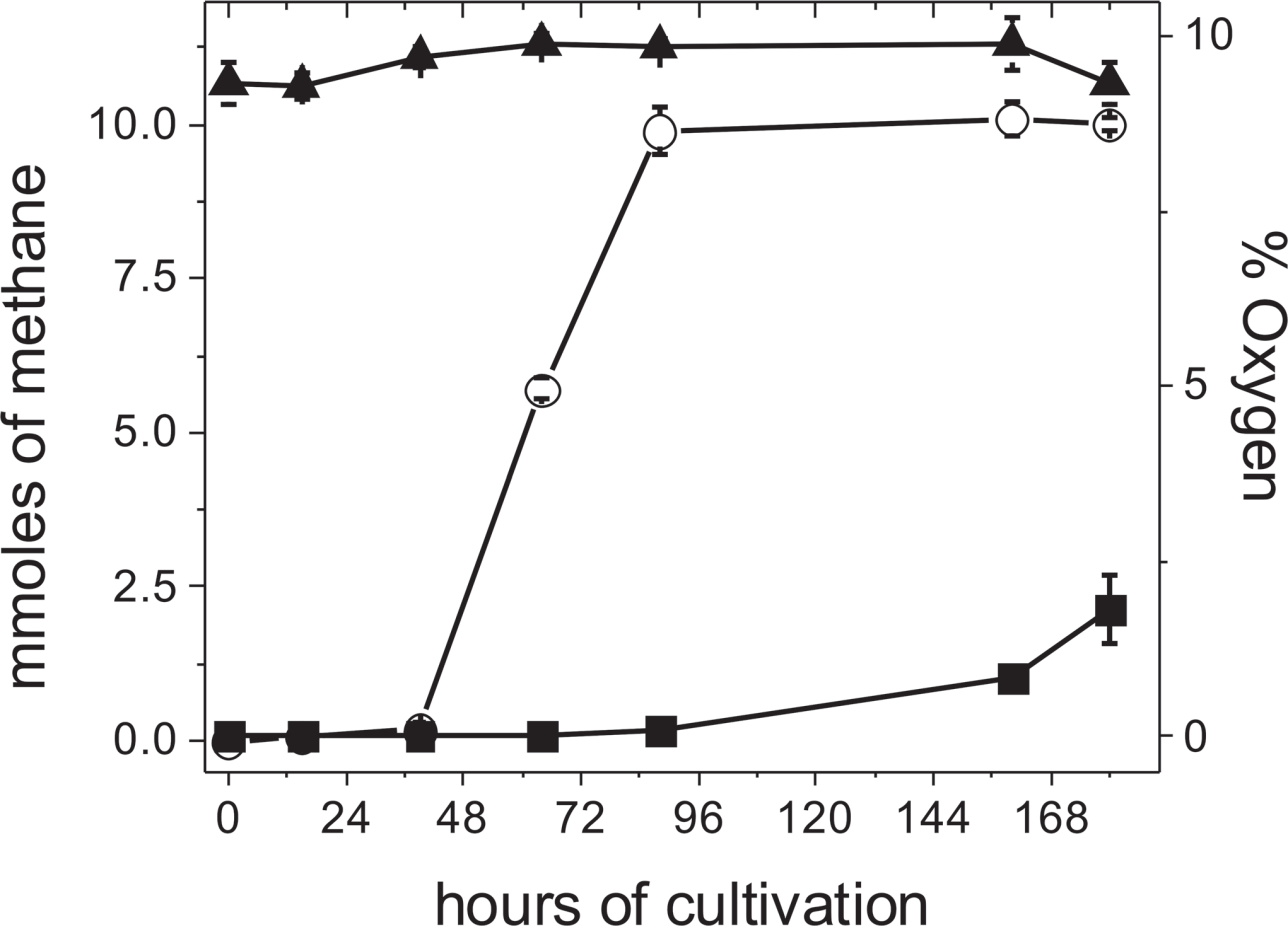
Effect of high O_2_ concentrations on methane production in *M*. *acetivorans*. Cells were cultured in 100 mL bottles with a syringe inserted in the rubber stopper to allow for gas exchange; the bottles were then incubated At 37°C in an 8 L anaerobic jar under steady 9% O_2_ (▲), as described in methods. The methane produced by anaerobic control (■) and air-adapted (○) cells throughout the growth curve was determined. Values are the mean ± SD of 4 bottles contained into the anaerobic jar.

The consumption of O_2_ by the non-enzymatic reaction with the sulfide and cysteine present in the culture medium did not significantly attenuate the O_2_ level reached in the head space even after the first air injection (Fig. 1C and 1D). In order to expose the cells to a higher and constant O_2_ level, culture bottles of control and air-adapted cells were also placed into an 8 liter home-made anaerobic jar containing 9% O_2_ (v/v), 73% N_2_ and 18% CO_2_. Due to the relatively high volume of the anaerobic jar and the high O_2_ concentration applied (29 mmol O_2_), no significant changes in the O_2_ concentration were determined throughout the cell growth timeframe (Fig. 2). Under these more severe oxidant conditions, air-adapted cells were able to generate methane at faster rates than control cells (Fig. 2). In fact, air adapted-cells consumed all the methanol added, with the concomitant production in 7.5 days of 10 ± 0.2 mmoles of methane/culture and 6.7 ± 0.8 mg protein /culture, whereas control cells produced only 2 mmoles of methane/culture and 2 ± 0.5 mg protein /culture (n = 4).

### 2.3 Effect of O_2_ on the contents of thiol-molecules and polyphosphates (PolyP)

The Cys and CoM-SH contents in air-adapted cells were similar to those in control cells; whereas their sulfide content increased significantly by 2.5–3.4 times (Table 2). Air-adapted cells grown on methanol or acetate showed a significant 2.2 or 5.3 fold increase in polyP content respect to anaerobic control cells. In addition, the Pi and polyP contents in acetate air-adapted cells were 2.5 and 5 times higher, respectively, than in methanol air-adapted cells (Table 2). The content of PolyP determined in methanol-grown cells was similar to that reported for different bacteria [21]. Increased polyP content was also visualized by the number of acidocalcisomes in air adapted cells in comparison with control anaerobic cells (S1 Fig.).

### 2.4 Oxidative stress damage and ROS production rates

Anaerobic control cells grown in the absence of O_2_ and further exposed to 2% (v/v) O_2_ for 2 h exhibited greater lipoperoxidation than air-adapted cells (S2A Fig.). Moreover, O_2_ exposure significantly increased ROS production with rates of 100 ± 46 and 2.1 ± 1.5 pmol (min x mg protein)^-1^ in control cells and air-adapted cells (n = 3), respectively (S2B Fig.). Further, the negligible ROS production in the absence of carbon source (methanol) suggested that cells have to be metabolically active to be able to produce ROS (S2B Fig.).

### 2.5 Antioxidant enzymes transcripts and activities

**Fig 3:**
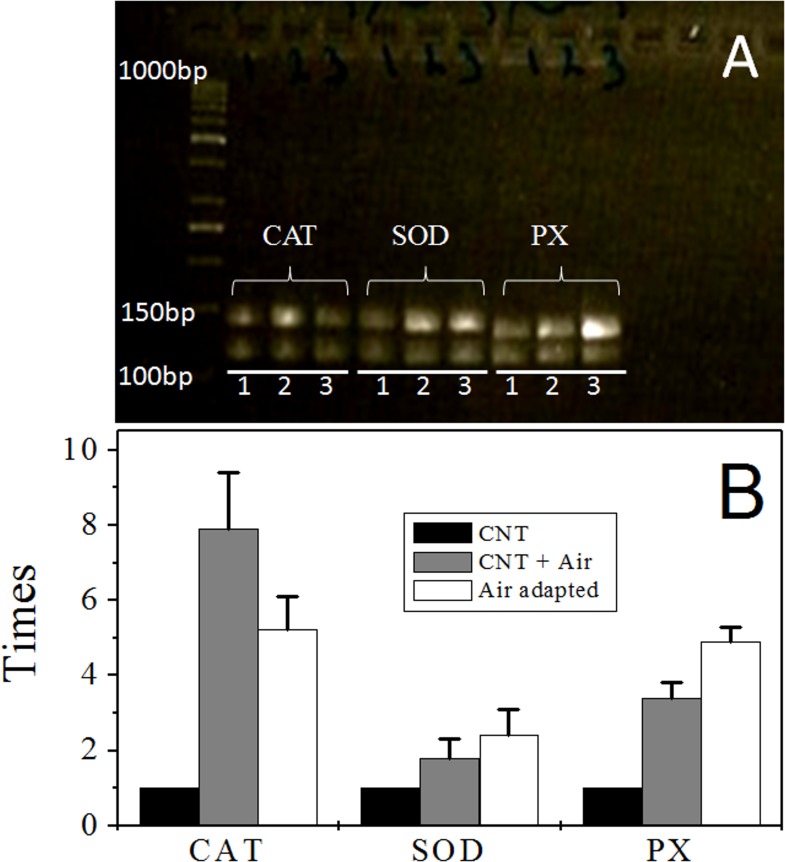
Transcript levels of anti-oxidant genes in *M*. *acetivorans*. Anaerobic control cells grown on acetate and harvested in the stationary phase were incubated at 37°C under orbital shaking in the absence (lane 1) or presence (lane 2) of 2% O_2_ for 2 h. Air-adapted cells grown and harvested in the same conditions were also exposed to O_2_ (lane 3). mRNA isolation and RT-PCR analysis was carried out by the primer dropping method as indicated in the Methods section and the PCR products separated by gel electrophoresis (A). Densitometric analysis (B) was carried out by double normalization *versus* the internal control MA3998 transcript and the target genes from anaerobic control cultures without O_2_ exposure (lane 1). Values are the mean of 4 independent experiments ± SD.

The analyses of the methanogen genomes sequenced so far showed that there are at least 10 different proteins with isoforms putatively involved in resistance mechanisms against oxygen (Table 1). Here, the canonical enzymes found in all three domains of life SOD, CAT and PXs were characterized. In order to determine whether transcripts of the genes annotated as SOD (MA1574), CAT (MA0972) and PX (MA1426) in *M*. *acetivorans* increase due to O_2_ exposure, 14 days-old acetate grown anaerobic control and air-adapted cells were exposed to 2% O_2_ for 2 h at 37°C, whereas a third set of strict anaerobic cells was maintained without O_2_ for comparison. Higher transcript levels for these enzymes were detected in cells exposed for the first time to 2% O_2_ for 2 h than in anaerobic control cells (2 ± 0.7, 8 ± 1 and 3.4 ± 0.4 times for SOD, CAT and PX, respectively). In air-adapted cells exposed to O_2_, higher transcript levels *versus* control cells were also determined (2.4 ± 0.5; 5.2 ± 1.5 and 5 ± 0.4 times for SOD, CAT and PX, respectively) (Fig. 3).

**Fig 4:**
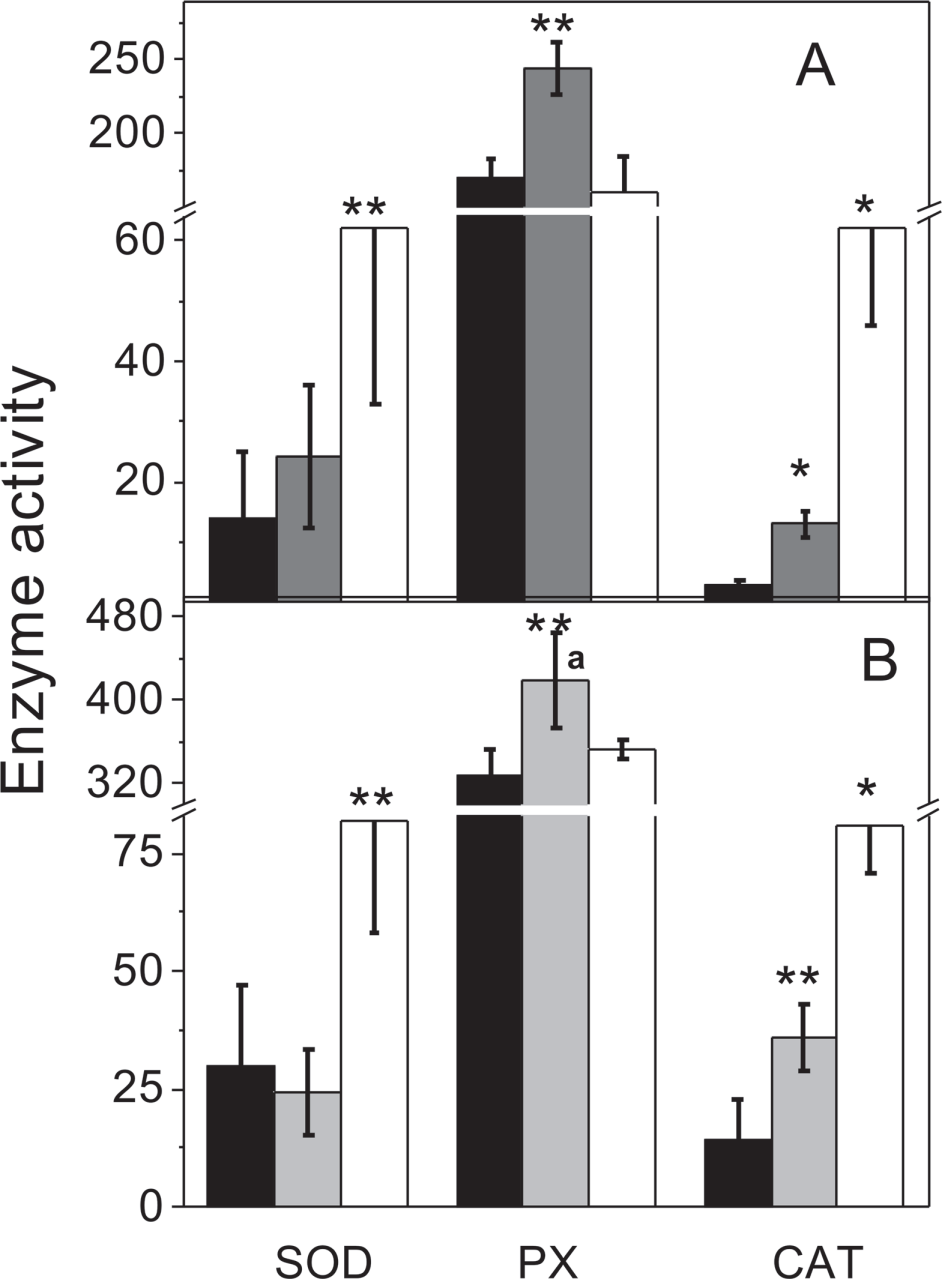
Antioxidant enzyme activities. Cytosolic-enriched fractions from anaerobic control (black bars), anaerobic *plus* 2% O_2_ for 2h (grey bars) and air-adapted cells (white bars) grown on methanol (A) or acetate (B) were used to determine activities of SOD, APX and CAT as described under Methods. Rate units for APX are mU (nmol of substrate consumed (min x mg protein)^-1^), whereas CAT and SOD activity units are U (mg protein)^-1^ as described under Methods. Values shown are the mean ± SD of at least 4 independent preparations. *P<0.01; ** P<0.05 *vs* anaerobic control cells. ^a^ P<0.01 *vs* methanol-grown cells.

Activities of SOD, CAT and PX were found in anaerobic-control cells, suggesting that there is a constitutively low level of activity for these enzymes, which might prevent any sudden oxidative damage when cells undergo episodes of O_2_ exposure or when their own cell metabolism produces ROS. However, in methanol and acetate cultures, air-adapted cells showed significant increases in SOD and CAT activities *versus* anaerobic control cells, whereas high PX activity (determined with ascorbate as electron donor) remained unchanged (Fig. 4). The acetate grown air-adapted cells showed significantly (2-times) higher PX activity than methanol-grown air-adapted cells (Fig. 4). SOD and CAT activity values were similar to those reported for *M*. *cuticularis* and *M*. *arboriphilus* [12].

Four genes annotated for PXs were found in the genome of *M*. *acetivorans* (MA1426, MA0993, MA2908 and MA0972) putatively specific for electron donors such as NAD(P)H, cytochrome *c* (cyt *c*) and catalase-type PX. After 2 h of incubation with 2% O_2_, increased PX activity with ascorbate as electron donor was apparent in control cells (Fig. 4; Table 3). With NADH and cyt *c*, significant increased PX activity was determined only for air adapted cells; CoM-SH a key metabolite in the methanogenic pathway served also as an electron donor for PX activity (Table 3).

**Table 3:** Different electron donors for *M*. *acetivorans* peroxidase activities.

Carbon source		NADH		NADPH		Reduced Cyt *c*		CoM-SH	Ascorbate
		**340 nm**	**Xyl-Or**	**340 nm**	**Xyl-Or**	**550 nm**	**Xyl-Or**	**Xyl-Or**	**Xyl-Or**
Methanol	CNT	188 ± 33	193 (2)	264 ± 23	270 (2)	218 ± 25	212 (2)	154 (2)	170 ± 13
	CNT+A	223 ± 21	235 ± 17	273 ± 19	280 ± 16	233 ± 17	242 ± 8	187 ± 12	244 ± 17^a^
	AA	238 ± 58	ND	259 ± 44	ND	286 ± 34^a^	ND	ND	160 ± 25
Acetate	CNT	505 ± 27	532 (2)	522 ± 36	504 (2)	323 ± 30	336 (2)	308 (2)	327 ± 26
	CNT+A	558 ± 39	571 ± 36	546 ± 89	524 ± 98	371 ± 38	387 ± 32	422 ± 17	419 ± 45^b^
	AA	573 ± 33^b^	ND	563 ± 38	ND	389 ± 25^b^	ND	ND	352 ± 19

CNT: control anaerobic cells; CNT+A: control anaerobic cells incubated with 2% O_2_ for 2h; AA: air adapted cells. Values are the mean ± SD of 3 independent experiments. ^a, b^ P<0.05 vs control anaerobic cells grown on methanol and acetate, respectively. ND, Not Determined; 340 nm indicates the activity determined by measuring consumption of NADH or NADPH; 550 nm indicates the activity determined by measuring the oxidation of cyt c; Xyl-Or indicates the activity determined by measuring the xylenol-orange complex formed by the remnant peroxide with xylenol.

Enzyme activities were also detected by protein gel electrophoresis. Regardless background and different electrophoretic performance for proteins proceeding from acetate and methanol grown cells, evident bands of activity were identified for SOD, CAT and PX (data not shown). In an attempt to determine the nature of the metal-cofactor dependence for SOD activity, Zn^2+^, Cu^2+^, Mn^2+^ and Fe^2+^ were tested; however, unmanageable background in the colorimetric essay surged with all metals tested, except for Zn^2+^, which activated 100% the SOD activity at 200 μM. Hence, the effect of 200 μM of each metal on SOD activity was determined by measuring the activity *in gel*, in which turbidity does not interfere. Thus, Zn^2+^, Cu^2+^, Fe^2+^ and Mn^2+^ showed a potent activating effect on SOD, whereas Cd^2+^ used as negative control, was innocuous (data not shown).

### 2.6 Structural analysis of air-adapted cells

**Fig 5:**
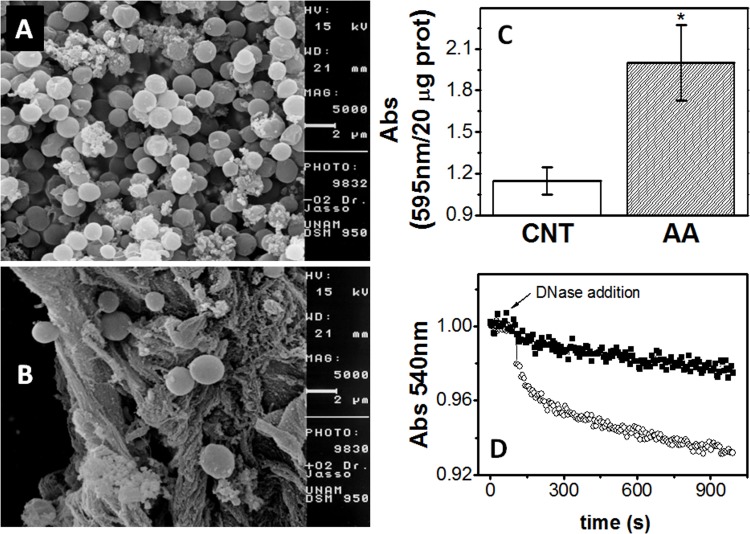
Formation of a biofilm extracellular matrix induced by O_2_ stress in *M*. *acetivorans*. Samples were prepared for and analyzed by scanning electron microscope as described in the Methods section. Extracellular matrix was absent in cells grown in the absence of oxygen (A) but well-defined in air-adapted cells (B). The carbon source was methanol, which was initially 100 mM and further replenished every 3 days. Micrographs are shown at 5000X, bar = 2 μm. (C) Biofilm formation determined by crystal violet staining in control-grown cells and air adapted cells cultured in methanol. *P < 0.01 *versus* control cells. (D) Extracellular DNA determination; 1 mg protein of control (filled squares) and air adapted cells (open circles) was added to a quartz cuvette with 1.8 mL of TME buffer. After 60 seconds of baseline acquisition, DNAse I was added and the light pass was determined at 540 nm.

It was recently demonstrated that *M*. *acetivorans* develops a biofilm as a protective mechanism against Cd^2+^ toxicity [22] and it is well established that O_2_ is also a triggering factor of biofilm formation in other microorganisms [23]. Hence, we hypothesized that the cell agglomerates, formed in air-adapted cell cultures after exposure to 2% (v/v) O_2_, were biofilms (S3A Fig.). To assess this hypothesis, structural analysis by scanning electron microscopy was used. Indeed, control cultures contained only planktonic cells (*i*.*e*. suspended cells; Fig. 5A, S3B Fig.). In contrast, agglomerations of methanol grown cells on the bottom of the air-adapted cell culture bottles developed after >18 days of 2% O_2_ exposure (S3A Fig.), constituted by an extracellular matrix to which cells appeared to be adsorbed (Fig. 5B).

To further show that the cell agglomerate in air adapted cells was indeed a biofilm and not a mere aggregation as it happens in *M*. *acetivorans* cultured in low salt medium [24], the crystal violet staining method to determine biofilm formation was used. Indeed, the ability of air adapted cells to form biofilm was significantly higher than that of control anaerobic cells (Fig. 5C). In addition, the chemical composition of the cell aggregates: protein, carbohydrates and extracellular DNA, essential components of biofilms [23], was also evaluated. The content of protein after 18–20 days did not change between control and air adapted cells (20–25 mg protein culture^−1^). However, non-soluble carbohydrates (CHOs) were significantly different between control and air-adapted cells: 10 ± 1 μg CHOs (mg protein) ^−1^ (n = 3) and 16 ± 3 μg CHOs (mg protein)^-1^ (*n* = 4; *P* < 0.05), respectively, *i*.*e*. there was a 60% higher CHO content in air adapted cells. Moreover, as judged by the decrease in the absorbance (biofilm disaggregation) induced by adding DNAse I, the extracellular DNA was also significantly higher in air adapted cells than in control cells (Fig. 5D). DNAse I test was also applied to control and air adapted cells cultured in low salt. Again, only air adapted cells showed the development of a biofilm (S4 Fig.).

## Discussion

### 3.1 Analysis of genes related to protection against oxidative stress in methanogenic genomes

The genomes analyzed here, indicated that *rubrerythrin* (a non-haem iron protein) is wide-spread among methanogens, and together with rubredoxin and the SOR activity, is necessary for a complete ROS detoxification system [16, 25] (Table 1). F_420_H_2_ oxidase catalyzes the reduction of O_2_ to water and may play an important role against oxidative stress in methanogens [26]. Genes encoding thioredoxins (Trx), well known proteins involved in oxidative stress handling, were present in all 27 genera analyzed. *Methanosarcina spp* possesses up to 8 different genes encoding Trx suggesting multiple metabolic roles for this protein [18]. On the other hand, *M*. *acetivorans* and *M*. *barkeri* are the only methanogens with genes annotated for quinol: *cyt bd* oxidase, which suggests that this enzyme may not have an important role against oxidative stress (Table 1).

Except for the NADH-peroxidase gene (which was only found in *M*. *acetivorans* among methanogens), genes for oxidative stress management found in bacteria (*E*. *coli*) showed high identity with those identified in *M*. *acetivorans*: cyt *c* peroxidase (30%), Cu-Zn SOD (34%), Fe-Mn SOD (40%) and catalase/peroxidase (60%). Archaea such as *Archaeoglobus sp* and *Methanobacterium sp* showed 61 and 80% identity (respect to *M*. *acetivorans* gene) for catalase/peroxidase, respectively. Fe-Mn SOD showed high identity (57–82%) among archaeal genomes. In contrast, *Pyrococcus spp* does not contain any of these enzymes coded in their genome.

### 3.2 Role of thiol-molecules and polyP as anti-oxidant metabolites

In air-adapted cells, the content of thiol-molecules increased 2 times *versus* anaerobic control cells (Table 2). If an intracellular volume of 0.7 μL (mg protein)^-1^ is assumed for *M*. *acetivorans* [27, 28], high concentrations of 70–114 mM and 7–14 mM may be reach up for Cys and CoM-SH, respectively. Hence, it is possible that this high Cys level directly reacts with O_2_ and ROS, and may induce the expression of antioxidant genes in response to oxidative stress such as Trx [29] or Rbr, Rbx, Prx and glutaredoxin-like proteins (Table 1). In turn, CoM-SH was an electron donor for PX activity (Table 3). We previously reported that the contents of Cys, CoM-SH and sulfide also increase (>7 times) in *M*. *acetivorans* exposed to Cd^2+^ respect to control cells without Cd^2+^ [22]. Therefore, an essential role for these two metabolites (Cys and CoM-SH) in the anti-oxidant machinery of this archaeon is proposed.

Increased synthesis of polyP is another mechanism in air-tolerant organisms involved in coping with different types of stresses such as heavy metals [30] and oxidative stress [31]. In the present work, it was described that air exposure triggered increased synthesis of polyP (Table 2; S1 Fig.), although acetate-grown cells accumulated more polyP than methanol-grown cells. Clearly, more work is needed to determine whether (i) there is an O_2_ threshold that triggers the polyP synthesis in *M*. *acetivorans* and whether (ii) Pi and polyP directly may react with ROS.

In bacteria, involvement of polyP in the resistance to oxidative stress has been shown. PolyP is essential for biofilm development, quorum sensing and virulence in bacteria [32, 33]. It has been suggested that polyP or polyP kinase regulate the transcription of genes involved in the stress oxidative response such as CAT and SOD in *E*. *coli* [34, 35]. Since *M*. *acetivorans* is a marine methanogen where acetate and phosphorus are present at low levels, it might be an evolutionary advantage for its survival to possess highly efficient mechanisms for uptake and storage of Pi.

### 3.3 ROS production and the effect of O_2_ on antioxidant enzyme transcripts and activities

*M*. *acetivorans* was able to consume O_2_, being higher in methanol than in acetate grown cells (Fig. 1), because higher O_2_ levels were used in methanol grown cells. Enzyme activities directly involved in O_2_ consumption were not determined in the present study, but *M*. *acetivorans* contains F_420_H_2_ oxidase and several ferredoxins, flavodoxins and iron-sulfur proteins which may react with oxygen [36]. ROS production was significantly higher in methanol (*versus* acetate) grown cells and anaerobic control cells (*versus* air-adapted cells; S2 Fig.) after the short-term (2 h) exposure to 2% O_2_. Lower SOD and CAT activities may be the reason for the higher ROS levels in these cells.

Basal activities of SOD and CAT as well as increased activities induced by oxidant stressors have been also found in *Methanosarcina barkeri* [13] and other methanogens [12, 37]. *M*. *acetivorans* has been identified as an archaeon with resistance to O_2_ [38] and hydrogen peroxide in short-term exposures [17], whereas other methanogens are extremely sensitive to O_2_ [10]. The absence of genes encoding antioxidant enzymes in the latter group may be the reason for their extreme sensitivity. Air-adapted cells showed significantly increased CAT and SOD activities. *In gel* enzyme activities indicated that Zn^2+^ and Fe^2+^ increase SOD activity, as reported for the *M*. *arboriphilus* enzyme [37]. There are genes annotated for Zn/Cu–dependent SOD (MA2422) and Fe/Mn-dependent SOD (MA1574) in the genome of *M*. *acetivorans*. On the other hand, the increase in the SOD transcript induced by O_2_ in air-adapted cells (2.4 times) correlated with the increased SOD activity (3.8 times), which may be further stimulated by heavy metal divalent cations.

On the other hand, there are 4 annotated genes encoding for PXs with putative specific electron donors: catalase/peroxidase (MA0972), chloride peroxidase (MA0993), NADH peroxidase (MA1426) and cytochrome *c* peroxidase (MA2908). Hence, PX activity determined here with ascorbate, a non-physiological electron donor in *M*. *acetivorans*, may have underestimated total PX activity. Hence, other electron donors were examined. Because chloride PX is involved in the detoxification of polychlorinated biphenyls pollutants rather than in oxidative stress [39], this activity was not determined. Instead, CoM-SH was tested as PX substrate because of its high physiological levels and to be a potential electron donor like glutathione. PX activities were found for NADH, NADPH, cyt *c* and CoMSH. Transcription of the NADH Px gene increased 5 times in air-adapted cells grown on acetate. However, all PX activities were high in both control and air adapted cells, suggesting that these enzyme activities may be constitutive and hence required for protecting the cell against basal levels of oxidative stress. CAT and SOD were in turn over-expressed in air adapted cells, indicating that these enzymes are involved in contending against acute oxidative stress generated by external stressors such as O_2_, as proposed by Pedone et al [20].

The presence of these antioxidant enzymes in *M*. *acetivorans* suggests that episodes of oxidative stress in the marine environment in which this archaeon grows may be recurrent. Indeed, changes in the O_2_ concentration occur during disturbances of the deep sea by earthquakes and other meteorological events [40].

### 3.4 Biofilm formation induced by O_2_ stress

Composition analysis of the cell agglomerates, in control and air adapted cultures grown on high or low salt, demonstrated that in all conditions non-soluble carbohydrates were present, with 10-fold higher levels in the low salt cultures, in which secretion of methanocondrioitin and S-layer is involved [24]. However, air adapted cells exhibited higher content of non-soluble CHOs and extracellular DNA, an essential component of biofilms (Fig. 5D). Our results showed that long exposure of *M*. *acetivorans* cultures to O_2_ (at least 18 days) led to the formation of biofilms, apparently as a strategy to gain resistance against higher O_2_ concentrations. The significant increase in the content of non-soluble carbohydrates in air adapted cells was, however, only 50% higher than that of control cells. By comparison, *M*. *acetivorans* cells exposed to 1.4 mM CdCl_2_ exhibit 800% increased content of non-soluble carbohydrates respect to control cells [22], suggesting that in *M*. *acetivorans* O_2_ is a weak biofilm-inducer.

In this regard, it has been documented that cells within biofilms show increased tolerance to stressful environmental conditions. For instance, the biofilm made by the archaeon *A*. *fulgidus* in metal-depleted medium is induced by non-physiological drastic changes of pH and temperature, high concentrations of metals or by addition of xenobiotics or O_2_. Essential metals sequestered within the biofilm stimulate the growth, suggesting that cells may produce biofilm as a mechanism for concentrating cells and attaching to surfaces, as a protective barrier and as a nutrient reservoir [41]. Due to the fact that similar biofilms are formed by other archaea, biofilm formation might be a common stress response mechanism within the *Archaea* domain [23].

Viable methanogens have been detected in dry, aerobic environments such as dry reservoir sediment, dry rice paddies and aerobic desert soils, suggesting that methanogens have mechanisms for long-term survival under various environmental stresses [42–44]. In *Methanosarcina barkeri*, desiccation and the synthesis of extracellular polysaccharide are indeed survival mechanisms against oxygen, probable because minimize oxygen diffusion into the cell [45]. Then, it is clear that to elucidate (i) the specific mechanisms that archaeal cells have developed to cope with O_2_; and (ii) the specific interactions between biofilm and cells, further studies are required.

In conclusion, the generation of stable cultures of air-adapted cells of *M*. *acetivorans* allowed to clearly determining (i) variation in the expression and activity of the anti-oxidant enzymes SOD, CAT and PXs; (ii) changes in thiol-molecule and polyP contents; and (iii) the development of biofilm. These cellular mechanisms are required to maintain the cell viability, which might become molecular targets for enhancing biogas production under oxidative stress conditions.

## Methods

### 4.1 Growth conditions

Fifty mL of high salt medium (HS-medium) [24] with 1.6 mM Cys and 1 mM sulfide was supplemented with 100 mM acetate or 70 mM methanol as carbon sources and poured in 100 mL serum-like bottles, sealed with a butyl rubber stopper and secured with an aluminum crimp collar. The media were autoclaved at 121°C for 30 min and let them cool down. Fresh cells of *Methanosarcina acetivorans* C2A strain (DSM 2834), previously isolated from marine sediments in the summer branch of Scripps Canyon near La Jolla, CA [46] and kindly provided by Prof. James G. Ferry (Pennsylvania State University, USA), were inoculated in the HS-medium under anoxic conditions (<0.3 μmol O_2_) in an anaerobic chamber (COY Lab products, Michigan, USA) and further incubated for the indicated times at 37°C without shaking. Growth was determined by measuring methane production and protein content.

### 4.2 Generation of air-adapted cells

To assess whether exposure to O_2_ triggers an effective cellular response against oxidative stress in *M*. *acetivorans*, two pulses of 2 ml of sterile air (0.4% O_2_ or 16 μmol total O_2_) at days 6 and 10 for acetate-cultures; or two pulses of 5 mL of sterile air (1% O_2_ or 41 μmol total O_2_) at days 2 and 4 for methanol-cultures were applied. The redox probe resazurin present in the culture media was slightly oxidized as revealed by turning and remaining pink for about 30 min after each air injection, indicating that the O_2_ added sufficed to create a microaerophilic environment (see Fig. 1C and 1D). No removal of air from the culture bottles was carried out after each addition; the presence of O_2_ was permanent throughout the growth curve (see Results section 2.2). Aliquots of these microaerophilic cell cultures were transferred to fresh media every 6 and 14 days for methanol and acetate grown cells, respectively, to initiate a new cell culture following the same protocol of O_2_ exposures. After 3 months using this regime, a third addition of air was always made at the beginning of each new cell culture. Thereafter, as judged by the constant growth and methane production, a stable air-adapted cell culture was obtained. Higher volumes of air (5 ml air for acetate and 10 mL for methanol cultures) were also tested but no reproducible results were achieved and cell cultures sometimes did not grow (data not shown).

### 4.3 Metabolite contents

Cells in the stationary growth phase (6 days for methanol and 14 days for acetate cultures) were harvested by centrifugation under anaerobic conditions. The cell pellet was gently re-suspended and washed once with 50 volumes of a solution containing 50 mM Tris, 20 mM MgCl_2_ and 2 mM EGTA at pH 7.2 (TME buffer). The washed pellet was resuspended in 1 mL of TME buffer.

Intracellular contents of Cys and CoM-SH were determined by HPLC and post-column derivatizing with DTNB (5, 5'-dithiobis-(2-nitrobenzoic acid), whereas sulfide was determined by the methylene blue colorimetric method as reported elsewhere [22].

Methane production was determined by gas chromatography (GC) in a Shimadzu GC2010 apparatus (Shimadzu; Kyoto, Japan) equipped with a capillary column HP-PLOT/U of 30 m length, 0.32 mm I.D. and 10 μm film (Agilent, USA), and flame ionization detector.

For determination of intracellular inorganic phosphate (Pi), washed cells were ruptured by applying 2 sonication pulses of 1 min at maximal output in a sonifier (Branson; CT, USA) and aliquots of the cell homogenate were taken for Pi determination. Intracellular polyP was determined in the cell homogenates after adding 3% ice-cold perchloric acid (PCA), strongly vortexed for 1 min, further incubated for 0, 60, 120 and 240 min at 90°C and centrifuged; the released Pi was determined in the supernatant aliquots. Based on the hydrolysis of the cyclic hexametaphosphate (NaPO_3_)_6_, which is usually a mixture of polymeric metaphosphates (Sigma, UK), the yield was not higher than 40% of the theoretical polyP added after 60 min of incubation. Longer incubation times did not increase the standard hydrolysis (data not shown). Instead, the Pi released in the cell extracts increased with the time and after 120 min of incubation was roughly the same than after 240 min. Pyrophosphate was also hydrolyzed by 15–20% under the same PCA/high temperature treatment (data not shown). Pi was quantified according to the methodology reported by [47] using 4-(methylamino) phenol hemisulfate *plus* sodium bisulfite as reducing agent. To estimate the polyP content, the Pi content determined in the sonicated cells (in which polyP is preserved) was subtracted from the Pi content determined in the PCA-treated cell homogenates (in which polyP is hydrolyzed to Pi).

### 4.4 O_2_, ROS and TBARS

Changes in the concentration of O_2_ in the head space were monitored by GC using the capillary column HP-MOLESIEVE of 30 m length, 0.32 mm I. D. and 25 μm film (Agilent, USA), and a thermal conductivity detector, and calculated by using a standard curve of oxygen. On the other hand, O_2_ dissolved in the culture medium was determined polarographycally at 35°C by using an oxymeter (YSI; OH, USA) equipped with a Clark-type electrode and placed inside the anaerobic chamber. A baseline was recorded with hypoxic TME buffer, which had been previously bubbled with a gas mixture (80% N_2_, 15% CO_2_ and 5% H_2_) inside the anaerobic chamber for 2 h. Thereafter, an aliquot of cell-free culture medium collected at the end of the cell growth curve was added and the O_2_ present was recorded. The signal of the O_2_ concentration was calibrated by using dithionite in air-saturated TME buffer.

The relative rate of ROS production in control and air-adapted cells was determined spectrophotometrically by measuring the oxidation of dichlorofluorescein diacetate (DCFDA). Briefly, inside the anaerobic chamber, ~1 mg of cell protein, 50 mM methanol as cell substrate and 250 μM DCFDA were mixed in TME buffer and poured into a 4 mL cuvette and sealed with a rubber stopper. After a baseline was attained, 4 mL of air (20% O_2_) were injected and DCFDA oxidation was monitored at 500 nm. The rate of ROS production was calculated using the molar extinction coefficient of DCF of 59.5 mM^-1^ cm^-1^.

For determination of the malondialdehyde (MDA) content, as indicator of lipid peroxidation levels, cultures of control anaerobic and air-adapted cells were harvested in the stationary phase, exposed to sterile air (2% O_2_) and incubated for 2 h at 37°C under orbital shaking (150 rpm). Thereafter, the cells were collected by centrifugation and washed with TME buffer under anoxic conditions. MDA was determined by reacting with thiobarbituric acid (TBARS) using 3–5 mg cell protein [48]. A standard curve was made with tetraethoxypropane; the reaction was linear up to 3 nmol TBARS.

### 4.5 Semi-quantitative RT-PCR analysis

Total RNA was extracted by using the RNeasy Protect Cell Mini Kit (Qiagen; Valencia, CA, USA) according to the manufacturer instructions. After verifying the RNA integrity by gel electrophoresis, 5 μg RNA was converted into cDNA with the Super Script First-Strand Synthesis System (Invitrogen; Carlsbad, CA, USA) and quantified. Changes in transcript levels were determined by semiquantitative reverse-transcriptase PCR reaction following the “primer dropping method” [49]. The PCR reactions (20 μL) contained 1X Pfu DNA polymerase buffer (Fermentas; Ontario, Canada), 2 mM MgCl_2_, 0.5 mM deoxyribonucleotide phosphates mix (Fermentas), 7 pmol each of the forward and reverse primers specific for each gene (SOD, CAT, PX; length primers were 20 bp, see S1 Table), 1 μg of cDNA and 1 unit of Pfu DNA polymerase (Fermentas). The PCR protocol was one cycle at 95°C for 5 min, followed by 20 cycles at 95°C for 1 min, 57.2°C for 1 min and 72°C for 2 min. The number of cycles for each target transcript was previously tuned-up to ensure that the amplified products were within the linear interval of amplification under this PCR protocol. Seven pmol of the forward and reverse primers of the loading control gene transcript MA3998 [7, 50] were added during amplification of the target genes to complete only 19 cycles.

For the internal control, it was previously determined the absence of competition for substrates and DNA polymerase with the target transcripts during the double amplification; *i*.*e*., the same band intensities of the amplified products should be attained in the single or double amplification reactions. In addition, it was verified that only one PCR product was obtained for each set of primers; the PCR product lengths were in the range of 115–139 bp (S1 Table). The samples were supplemented with loading buffer and separated by standard DNA electrophoresis in a 2.5% agarose gel. Densitometric analysis was carried out and a double normalization was done using the MA3998 as loading control of the PCR reaction and against control cells not exposed to O_2_.

### 4.6 Enzyme activities

Cell samples from cultures in the stationary phase were re-suspended in lysis buffer containing 0.1 M sodium phosphate and 1 mM EDTA at pH 8.0 and broken by vigorous vortexing and incubating for 30 min at 4°C as reported previously [51]. The resulting cell homogenate was treated with DNAse I to eliminate viscosity. The entire procedure of preparing cell homogenates was carried out under anaerobic conditions.

For protein determination, samples were incubated overnight at 4°C with 3% TCA and further centrifuged to discard remaining resazurine, which interferes with the protein determination assay. The pellet was resuspended in water and protein was determined by the Lowry method using bovine serum albumin as standard.

CAT activity was determined at 35°C in 50 mM HEPES, 120 mM KCl, 1 mM EGTA and 50 mM NaCl at pH 7.0 (HKE-Na buffer) and 0.01–0.03 mg cell homogenate protein/mL. The reaction was started by adding 20 mM hydrogen peroxide and its consumption was monitored at 240 nm. An extinction coefficient of 43.6 M^-1^ cm^-1^ at pH 7.0 was used. One unit of enzyme activity is defined as 1 μmol of substrate catalyzed min^-1^. The activity was fully inhibited by 10 mM NaCN.

PX activity was determined at 35°C by the method of Jiang et al [52]. Different electron acceptors were tested: 1 mM NADH, 1 mM NADPH, 55 μM reduced cytochrome *c*, 5 mM CoM-SH or 10 mM ascorbate and 0.2–0.3 mg cell homogenate protein were mixed in HKE-Na buffer. The reaction was started by adding 50 μM cumene hydroperoxide. To stop the reaction, aliquots of the reaction assay taken at different times were mixed with 0.1 mM xylenol orange, 0.25 mM Fe(NH_4_)_2_(SO_4_)_2_, 100 mM sorbitol and 25 mM H_2_SO_4_. Remnant hydroperoxide reacted with xylenol orange to form a complex that was detected at 560 nm (ε = 26.9 mM^-1^ cm^-1^). The PX activity was linear for at least 1 min for all electron donors One milliunit of activity is equivalent to one nmol substrate consumed (min)^-1^. A slight absorbance change was detected in the absence of cell sample, which was subtracted. Also, no activity was detected in the absence of ascorbate. Dependence of NAD(P)H and cyt *c* on peroxidase activity was also determined spectrophotometrically at 340 nm and 550 nm, using the molar absorptivity coefficients (ε) of 6.22 mM^-1^ cm^-1^ and 21.1 mM^-1^ cm^-1^, for NAD(P)H and reduced cytochrome c, respectively.

Superoxide dismutase activity (SOD) was determined by a competitive inhibition assay using xanthine–xanthine oxidase system to reduce nitroblue tetrazolium (NBT). The reaction mixture contained 49 mM Na_2_CO_3_, 0.122 mM EDTA, 30.6 mM NBT, 0.12 mM xanthine, 0.06 mg free-fatty acid bovine serum albumin/ mL, 2.5–5 μg cell homogenate protein/0.1 mL and 2.5 mU xanthine oxidase/mL. The production of NBT-formazan was recorded at 560 nm. The amount of protein that inhibited NBT reduction by 50% was defined as one unit of SOD activity. Results were expressed as U/mg protein [53]. Several metals were tested as enzyme activators, but the intense background obtained, except for zinc, affected the assay (see results section). No activity was found in the absence of the xanthine–xanthine oxidase system.

SOD, CAT and PX activities were also determined *in gel*. 20 μg cell homogenate protein were subjected to native polyacrylamide gel electrophoresis (10% acrylamide for SOD and CAT and 8% for APX) and activities were revealed following pre-established protocols (See S1 Text file for further details).

### 4.7 Scanning transmission electron microscope (STEM) and biofilm components

After 6 days of growth, cultures were supplemented with 100 mM methanol *plus* 10 mL sterile air (2% O_2_) every 3 days until day 18. This treatment produced cell agglomerates (biofilms) in air-adapted cell cultures, but not in control anaerobic cell cultures. The precipitated cell agglomerates were separated from both the medium and planktonic cells by decantation and fixed with 2.5% (v/v) glutaraldehyde in TME buffer for its posterior preparation for electron microscopy as reported previously [51]; aliquots of cells from control cultures were centrifuged, resuspended in a small volume of TME buffer and also fixed with glutaraldehyde. For STEM, the fixed cells were post-fixed with 1% osmium tetroxide in TME for 2 h, and then rinsed three times with the same buffer. Each cell sample was placed in a filtration system with a 13 mm polycarbonate membrane of 0.6 μm diameter pore (Whatman; Kent, UK) and dehydrated with increasing concentrations of ethanol. Each membrane was recovered and dried in a critical point dryer apparatus (Polaron E5000; West Sussex, UK) with carbon dioxide. The samples were mounted on aluminum stubs on carbon double-side sticker and covered with 10 nm ionized gold film using coater system equipment (Polaron 11-HD). Finally, the samples were analyzed in a scanning electron microscope (Zeiss DSM-950; Oberkochem, Germany) with secondary electrons accelerated at 15 kV.

In parallel, a second sample of biofilm was resuspended in TME buffer and homogenized. The biochemical composition of the biofilm, protein, carbohydrates and DNA was determined as reported elsewhere [22].

In turn, biofilm formation was followed by the crystal violet staining method which is widely used to determine static biofilm formation in bacteria [54] and methanogens [22]. Briefly, control and air adapted cells were cultured for 3 days in fresh medium in 96-well polystyrene plates and incubated at 37°C in an anaerobic jar filled with a mixture of 73% N_2_, 18% CO_2_ and 9% O_2_ (V/V) by using a multiple flow tube rotameter (Daigger, Ill, USA). Thereafter, 0.1% (w/v) crystal violet was added and incubated for 20 min; two washing steps with TME buffer were carried out and addition of absolute ethanol for dye solubilization was made; hence, the optical density of the crystal violet retained by the cells was recorded at 595 nm.

### 4.8 Exposure and tolerance to oxic conditions

To assess whether adapted cells indeed acquired enhanced skills to resist extreme oxic conditions, 4 culture bottles (with sterile needles through the stopper to allow for gas exchange between the bottle and the anaerobic jar) each of air adapted or control cells were placed into an 8 liter anaerobic jar. The 8 L jar cap equipped with a rubber stopper, was sealed with a heat resistant silicone sealant and filled with a mixture of 73% N_2_, 18% CO_2_ and 9% O_2_ (V/V). Hence, samples of gas were taken at different times to determine the rates of O_2_ and methane production by the 4 cell culture bottles. After 7.5 days, the anaerobic jar was uncapped and the cells in the 4 bottles were harvested by centrifugation and the protein content of each bottle was determined.

### 4.9 Genome analysis

Methanogenic genomes were obtained from the Kyoto Encyclopedia of Genes and Genomes (KEGG) online database (http://www.kegg.jp/kegg/catalog/org_list.html). Genomes from 27 genera published so far were analyzed. Strain types were selected where possible and incomplete sequences were ignored. The genomes analyzed are shown in Table 1.

## Supporting Information

S1 Fig Electron microscopy analysis of *Methanosarcina acetivorans*. HAADF-STEM projection images of air adapted cells (A) and control anaerobic cells (B), cultured in methanol. Enclosed in dashed circles, cell in the image (A) revealed high amounts of electro-dense dark granules (acidocalcisomes) surrounding the internal cell membrane (indicated by arrows), whereas in (B) these granules were scarce. Elemental analysis of these granules (C) showed high amounts of P, Ca and Al indicating that the acidocalcisomes were indeed filled with PolyP. Bar for air adapted cells: 0.5 μm; for control cells: 0.2 μm.
(TIF)

S2 Fig Lipoperoxidation in *M*. *acetivorans*. (A) MDA content was determined in anaerobic control and air-adapted cells grown on methanol (white bars) or acetate (black bars) after 2 h of adding 2% O_2_ as described in methods. Values are the mean ± SD of at least 3 independent preparations. *P<0.01 *vs* anaerobic control cells. (B) Representative traces of direct ROS production driven by O_2_ addition in methanol-grown cell suspensions (see methods section for details). Trace 1: anaerobic-control cells, trace 2: air-adapted cells, trace 3: anaerobic-control cells without methanol as substrate, trace 4: anaerobic-control cells *plus* 0.2 mM cysteine where ROS was not detected. Underlined numbers on the traces indicate the rate of ROS production in pmol ROS produced (min x mg cellular protein)^-1^.
(TIF)

S3 Fig Cell aggregates formation induced by O_2_ in cultures of *M*. *acetivorans* with methanol. Representative pictures of cultures grown in the presence (A) or absence of 2% (V/V) of O_2_ (B). It is noted that control cultures without air injected did not develop cell aggregates, whereas air adapted culture cells showed cell aggregates. See section 2.6 of results for more details.
(TIF)

S4 Fig Effect of DNAase I on the turbidity of *M*. *acetivorans* cell suspensions cultured in low salt medium. Absorbance changes of one mg protein from three independent cell cultures under 0.1 M NaCl are shown: control cells (filled symbols) and air adapted cells (open symbols) were added to a quartz cuvette with 1.8 mL of TME buffer. After 60 seconds of baseline acquisition, DNAse I was added and the light pass was determined at 540 nm.
(TIF)

S1 Table Sequences of the primers used for identification of the transcripts. (DOCX)

S1 Text Methods and results. (DOC)
