# Supplementary figures and images for: Air-Adapted Methanosarcina acetivorans Shows High Methane Production and Develops Resistance against Oxygen Stress
Source: PLoS One. 2015 Feb 23;10(2):e0117331. doi: 10.1371/journal.pone.0117331 (PMC4338226; doi:10.1371/journal.pone.0117331)

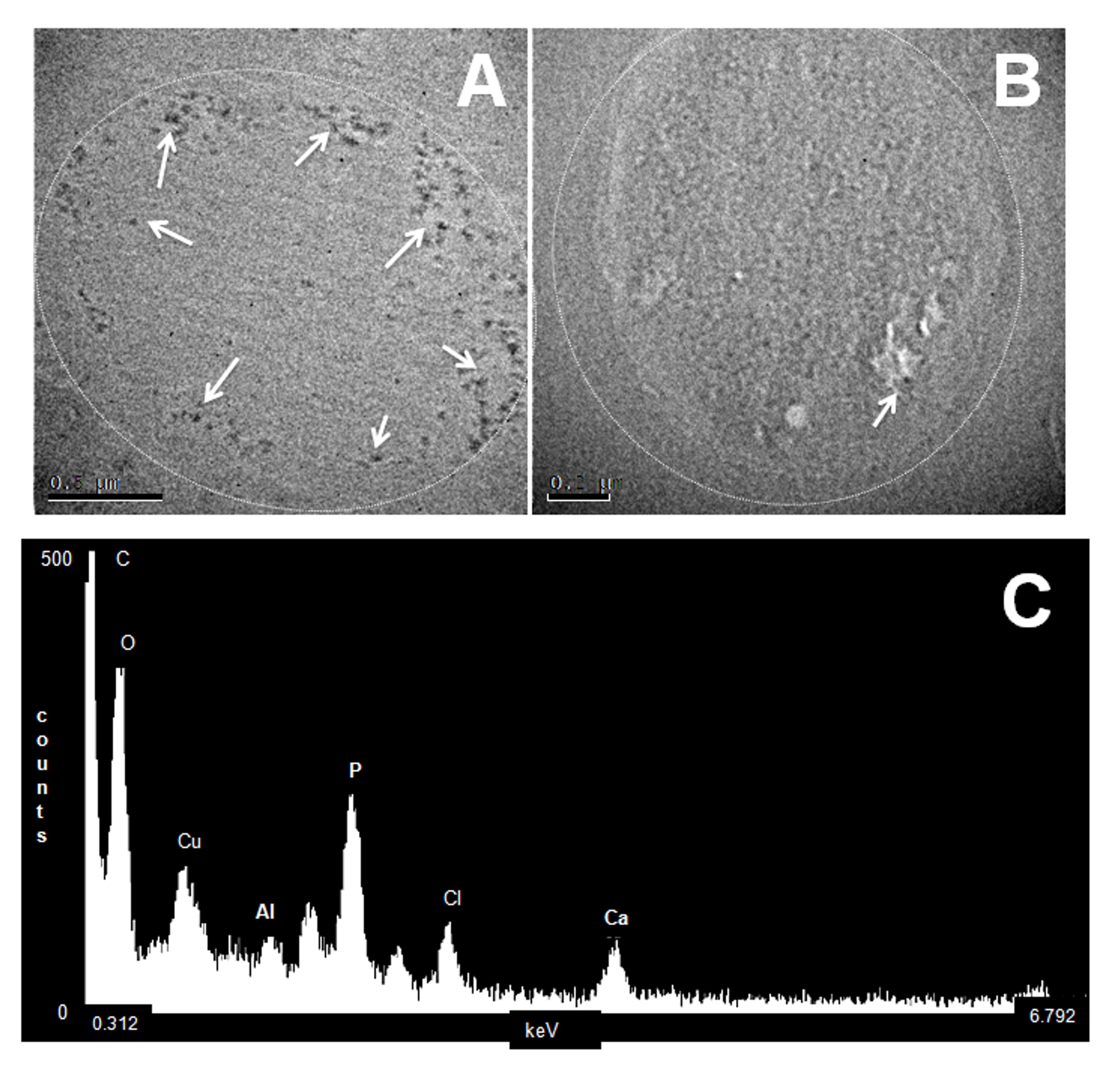

Supplement: S1 Fig — HAADF-STEM projection images of air adapted cells (A) and control anaerobic cells (B), cultured in methanol. Enclosed in dashed circles, cell in the image (A) revealed high amounts of electro-dense dark granules (acidocalcisomes) surrounding the internal cell membrane (indicated by arrows), whereas in (B) these granules were scarce. Elemental analysis of these granules (C) showed high amounts of P, Ca and Al indicating that the acidocalcisomes were indeed filled with PolyP. Bar for air adapted cells: 0.5 μm; for control cells: 0.2 μm. (TIF) [file pone.0117331.s001.tif]

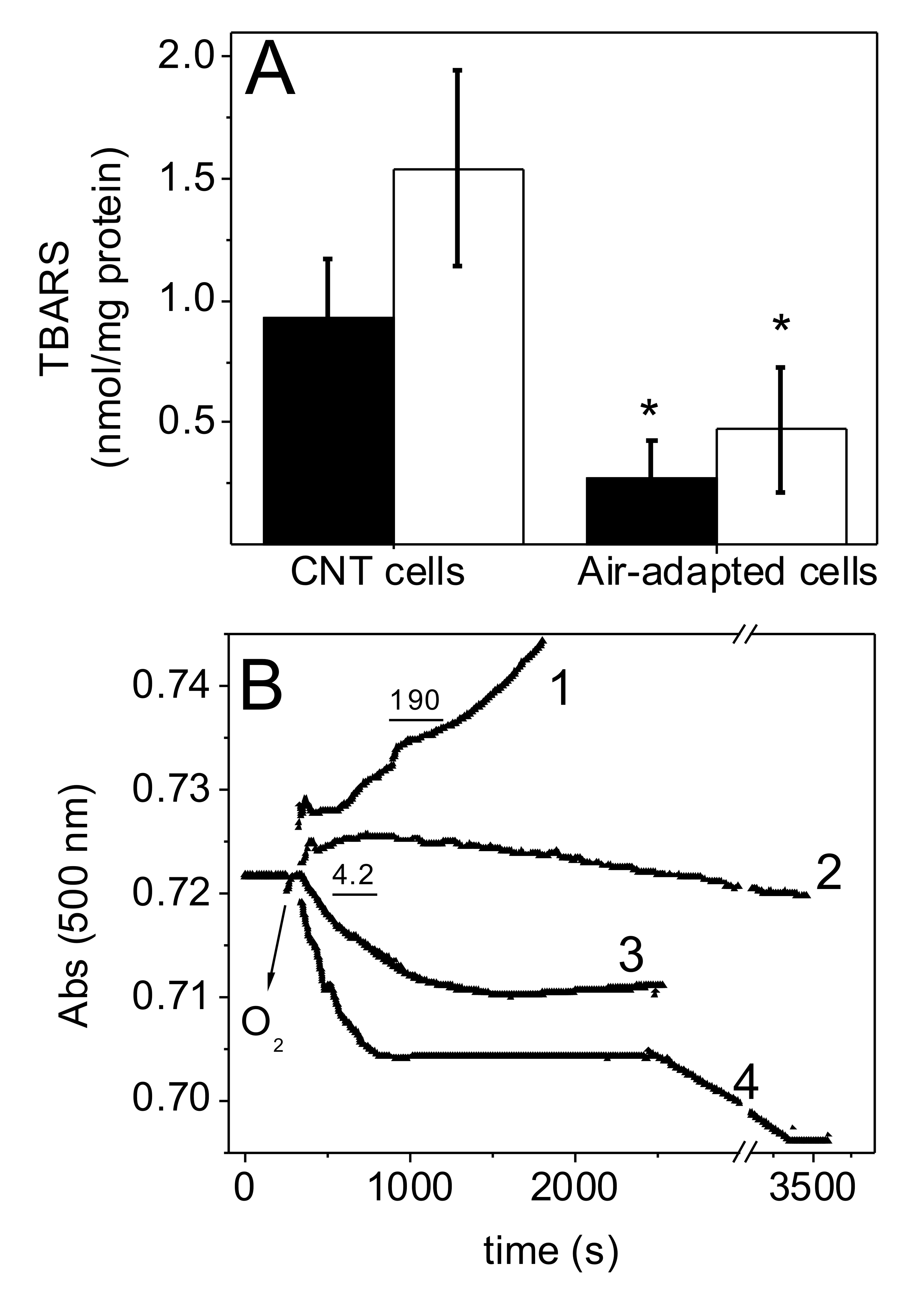

Supplement: S2 Fig — (A) MDA content was determined in anaerobic control and air-adapted cells grown on methanol (white bars) or acetate (black bars) after 2 h of adding 2% O2 as described in methods. Values are the mean ± SD of at least 3 independent preparations. *P<0.01 vs anaerobic control cells. (B) Representative traces of direct ROS production driven by O2 addition in methanol-grown cell suspensions (see methods section for details). Trace 1: anaerobic-control cells, trace 2: air-adapted cells, trace 3: anaerobic-control cells without methanol as substrate, trace 4: anaerobic-control cells plus 0.2 mM cysteine where ROS was not detected. Underlined numbers on the traces indicate the rate of ROS production in pmol ROS produced (min x mg cellular protein)-1. (TIF) [file pone.0117331.s002.tif]

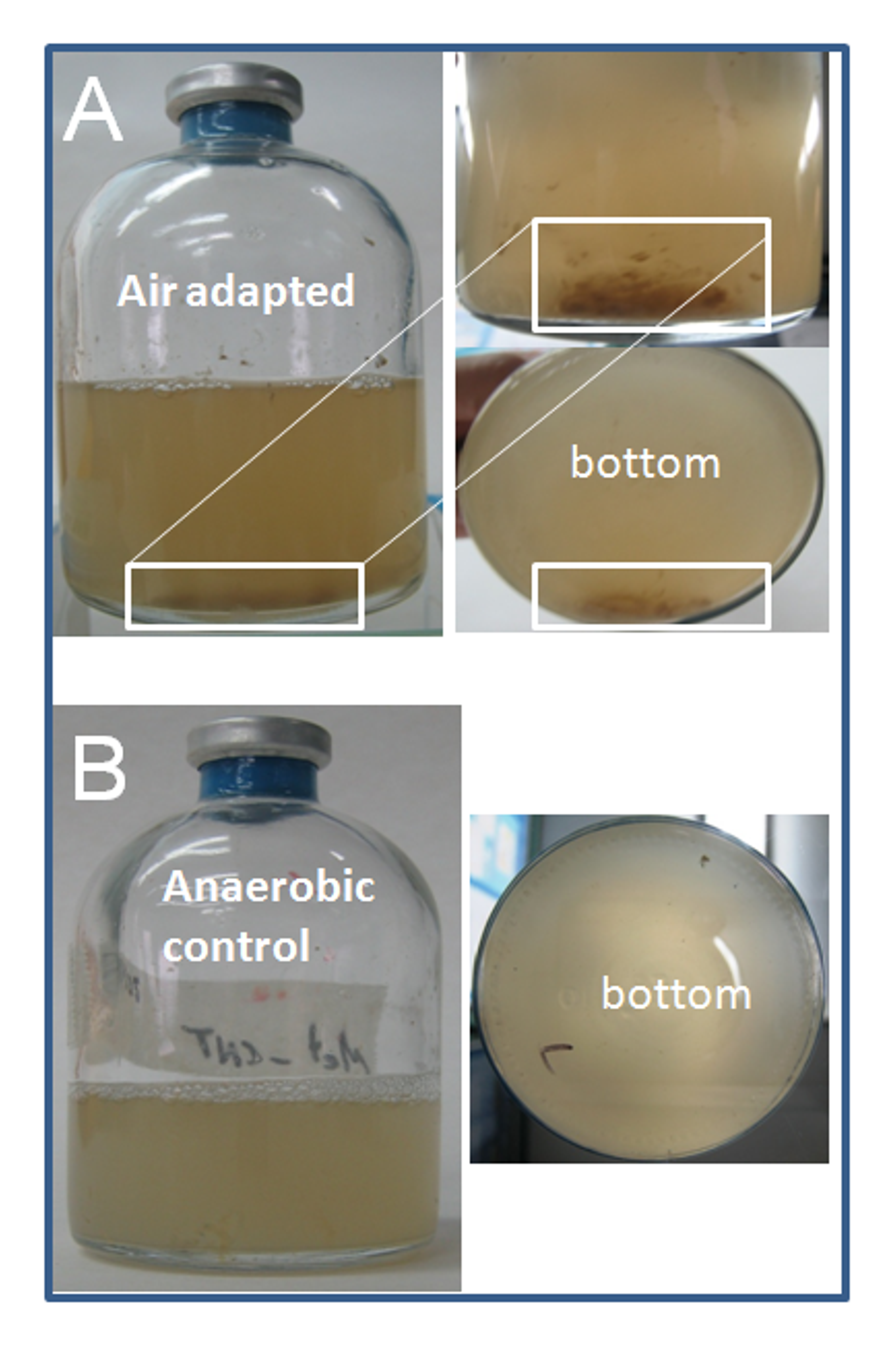

Supplement: S3 Fig — Representative pictures of cultures grown in the presence (A) or absence of 2% (V/V) of O2 (B). It is noted that control cultures without air injected did not develop cell aggregates, whereas air adapted culture cells showed cell aggregates. See section 2.6 of results for more details. (TIF) [file pone.0117331.s003.tif]

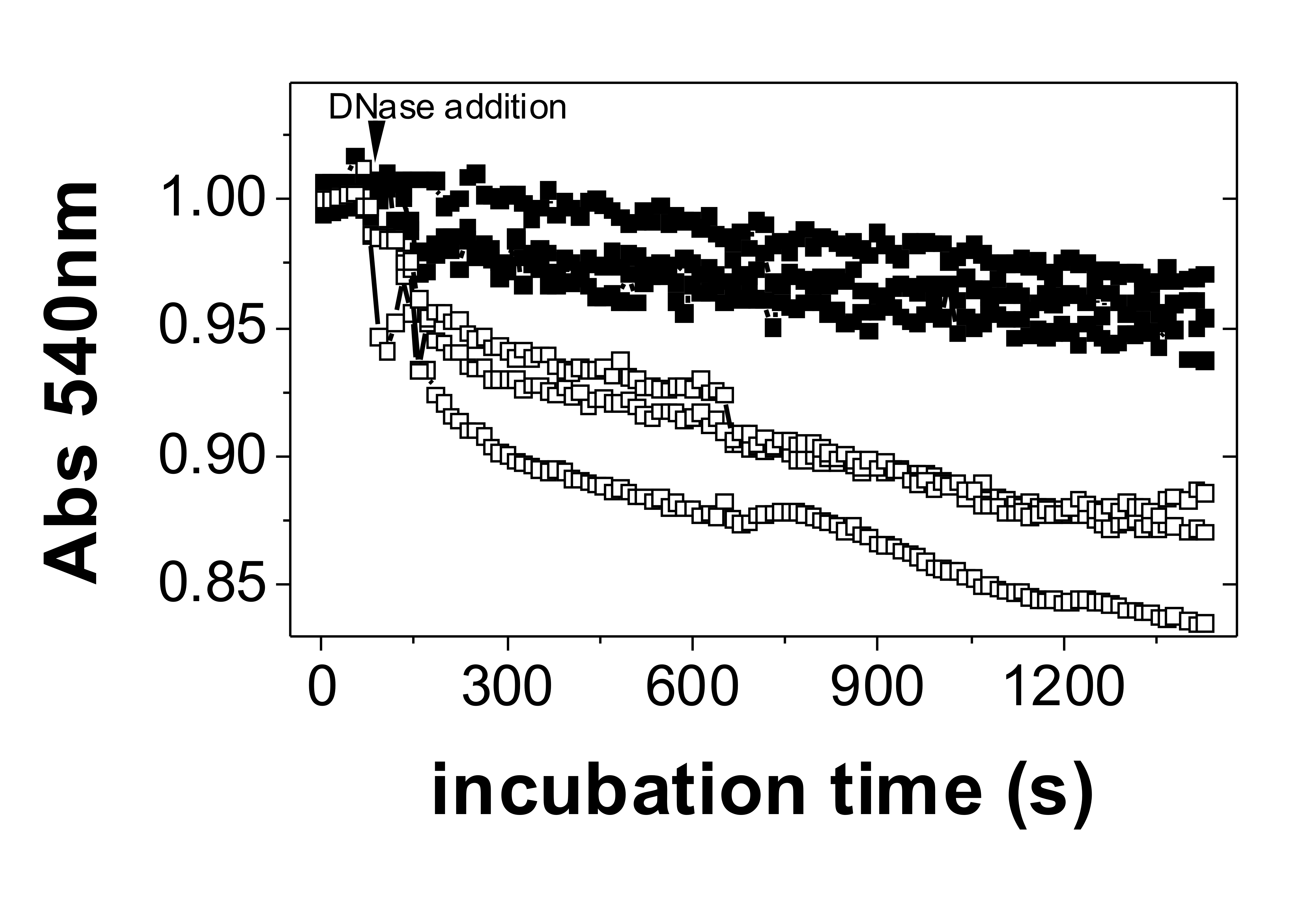

Supplement: S4 Fig — Absorbance changes of one mg protein from three independent cell cultures under 0.1 M NaCl are shown: control cells (filled symbols) and air adapted cells (open symbols) were added to a quartz cuvette with 1.8 mL of TME buffer. After 60 seconds of baseline acquisition, DNAse I was added and the light pass was determined at 540 nm. (TIF) [file pone.0117331.s004.tif]
