# Supplementary material for: Air-Adapted Methanosarcina acetivorans Shows High Methane Production and Develops Resistance against Oxygen Stress
Source: PLoS One. 2015 Feb 23;10(2):e0117331. doi: 10.1371/journal.pone.0117331 (PMC4338226; doi:10.1371/journal.pone.0117331)
Supplement: S1 Table — (DOCX) [file pone.0117331.s005.docx]

**Table S1. Sequences of the primers used for identification of the transcripts**

| **gene** | **Forward primer sequence** | **Reverse primer sequence** | **PCR product lengths (bp)** |
| --- | --- | --- | --- |
| SOD (MA1574) | 5´CCCTTCTGGAAATGATGGAC3´ | 5´TTGCTTGCAGGAGTCATCTC3´ | 139 |
| CAT (MA0972) | 5´CGGAGCAAACAAACAAGAAA3´ | 5´TGAACTCCTTCGCGTAATTG3´ | 132 |
| PX (MA1426) | 5´AAAGCAGCCGTAAATGTTCC3´ | 5´GAAGTCAGGTTCGCTGATGA3´ | 132 |
| Reference gene (MA3998) | 5´TGATCGAGAAACTGGCAGAC3´ | 5´TGCCTGACCATGGATACACT3´ | 115 |
