# Supplementary material for: Air-Adapted Methanosarcina acetivorans Shows High Methane Production and Develops Resistance against Oxygen Stress
Source: PLoS One. 2015 Feb 23;10(2):e0117331. doi: 10.1371/journal.pone.0117331 (PMC4338226; doi:10.1371/journal.pone.0117331)
Supplement: S1 Text — (DOC) [file pone.0117331.s006.doc]

Supplementary materials

**Methods**

**Enzyme activities in gel**

Superoxide dismutase (SOD), CAT and peroxidase (APX) activities were determined in native protein gel electrophoresis following pre-established protocols [1-3]. Twenty µg of cell homogenate protein were subjected to native polyacrylamide gel electrophoresis (PAGE) (10 % acrylamide for SOD and CAT and 8 % for APX). For in-gel SOD activity assays, the protein separation was carried out at 90 V for 4 h at 4°C. Afterwards, the gels were washed and then incubated with 50 mM sodium phosphate buffer (pH 7.8) *plus* 0.028 mM riboflavine and 28 mM TEMED (N,N,N',N'- tetramethylethylenediamine) for 30 min under gentle shaking. After washing with distilled water, the gels were soaked into the phosphate buffer adding 2.5 mM NBT (nitro blue tetrazolium) and a metal mix (0.5 mM CuSO_4_, 0.5 mM ZnSO_4_, 0.5 mM CoCl_2_ and 0.5 mM FeCl_3_) for 2 h under gentle shaking, irradiating UV light until bands became visible. For PX activity, the gel was incubated in the same 50 mM phosphate buffer pH 7.4 *plus* 4 mM ascorbate and 2 mM H_2_O_2_ for 30 min. After washing with water, activity was revealed by adding 28 mM TEMED and 2.4 mM NBT under UV light for 10-20 min. For CAT, the samples were separated by 8 % PAGE; then, the gels were washed with 50 mM sodium phosphate buffer (pH 6.4) and further incubated with 50 mM sodium phosphate buffer (pH 6.4) *plus* 0.02 % 3,3´-diaminobenzidine (w/v) and 0.006 % hydrogen peroxide (v/v) for 30 min under gentle shaking. After washing with distilled water, the gels were soaked in 0.006% hydrogen peroxide for 10 min under gentle shaking. After further washing with water, gels were stained with a solution of 1 % potassium ferricyanide (w/v) and 1 % ferric chloride (w/v) until bands became visible under UV light. The gels were further washed with water and photographed. Proteins in the gel were also stained with Coomassie brilliant blue to verify that protein loads were similar.

**Results**

**Chemical composition of biofilm developed in air adapted cells cultured at low salt.**

*M. acetivorans* cultured in low salt (0.1 M NaCl) media showed a protein content of 20 ± 1.7 and 17.5 ± 2.3 mg protein (culture)^-1^ for control and air adapted cultures, respectively. In turn, cultures in low salt showed 104 ± 6 and 170 ± 16 µg CHO´s (mg protein)^−1^ for control and air adapted cells, respectively (n = 5; mean ± SD; *P* < 0.01), *i.e.* there was a 63 % higher CHO content in air adapted cells. These values in the external CHO content for both control and air adapted cells grown in low salt are one order of magnitude higher than in high salt grown cells (section 2.6 of results). Similarly, a 10-times increase in total CHOs in control cells in low salt *versus* high salt was previously determined for glucuronic acid, the main component of methanocondroitin in *Methanosarcina* sp [4]. Extracellular DNA was also found in the air adapted cells cultured in low salt, as judged by the decrease in the absorbance (biofilm disaggregation) by addition of DNAse I (Fig. S4).

References

1. [Beauchamp C](http://www.ncbi.nlm.nih.gov/pubmed?term=Beauchamp C%5BAuthor%5D&cauthor=true&cauthor_uid=4943714), [Fridovich I](http://www.ncbi.nlm.nih.gov/pubmed?term=Fridovich I%5BAuthor%5D&cauthor=true&cauthor_uid=4943714) (1971) Superoxide dismutase: improved assays and an assay applicable to acrylamide gels. [Anal Biochem](http://www.ncbi.nlm.nih.gov/pubmed/?term=Beuchamp%2C+Fridovich%2C+1971) 44: 276-287.

# Mittler R, Zilinskas BA (1993) Detection of ascorbate peroxidase activity in native gels by inhibition of the ascorbate-dependent reduction of nitroblue tetrazolium. [**Anal Biochem**](http://www.ncbi.nlm.nih.gov/pubmed/?term=-%09Mittler+R%2C+Zilinskas+B.+(1993).) 212: 540-546.

1. Yamashita K, Shiozawa A, Watanabe S, Fukumori F, Kimura M, Fujimura M (2008) ATF-1 transcription factor regulates the expression of ccg-1 and cat-1 genes in response to fludioxonil under OS-2 MAP kinase in *Neurospora crassa*. Fungal Gen Biol 45: 1562-1569.
2. Sowers KR, Boone JE, Gunsalus RP (1993) [Disaggregation of *Methanosarcina* spp. and growth as single cells at elevated osmolarity.](http://www.ncbi.nlm.nih.gov/pubmed/16349092) Appl Environ Microbiol 59: 3832-3839.

Figure legends

Figure S1. Electron microscopy analysis of *Methanosarcina acetivorans*

HAADF-STEM projection images of air adapted cells (A) and control anaerobic cells (B), cultured in methanol. Enclosed in dashed circles, cell in the image (A) revealed high amounts of electro-dense dark granules (acidocalcisomes) surrounding the internal cell membrane (indicated by arrows), whereas in (B) these granules were scarce. Elemental analysis of these granules (C) showed high amounts of P, Ca and Al indicating that the acidocalcisomes were indeed filled with PolyP. Bar for air adapted cells: 0.5 µm; for control cells: 0.2 µm.

# Figure S2. Lipoperoxidation in *M. acetivorans*

# (A) MDA content was determined in anaerobic control and air-adapted cells grown on methanol (white bars) or acetate (black bars) after 2 h of adding 2% O_2_ as described in methods. Values are the mean ± SD of at least 3 independent preparations. *P<0.01 *vs* anaerobic control cells. (B) Representative traces of direct ROS production driven by O_2_ addition in methanol-grown cell suspensions (see methods section for details). Trace 1: anaerobic-control cells, trace 2: air-adapted cells, trace 3: anaerobic-control cells without methanol as substrate, trace 4: anaerobic-control cells *plus* 0.2 mM cysteine where ROS was not detected. Underlined numbers on the traces indicate the rate of ROS production in pmol ROS produced (min x mg cellular protein)^-1^

Figure S3. Cell aggregates formation induced by O_2_ in cultures of *M. acetivorans* with methanol.

Representative pictures of cultures grown in the presence (A) or absence of 2% (V/V) of O_2_ (B). It is noted that control cultures without air injected did not develop cell aggregates, whereas air adapted culture cells showed cell aggregates. See section 2.6 of results for more details.

Figure S4. Effect of DNAase I on the turbidity of *M. acetivorans* cell suspensions cultured in low salt medium.

Absorbance changes of one mg protein from three independent cell cultures under 0.1 M NaCl are shown: control cells (filled symbols) and air adapted cells (open symbols) were added to a quartz cuvette with 1.8 mL of TME buffer. After 60 seconds of baseline acquisition, DNAse I was added and the light pass was determined at 540 nm.

**Table S1**. Sequences of the primers used for identification of the transcripts

| **gene** | **Forward primer sequence** | **Reverse primer sequence** | **PCR product lengths (bp)** |
| --- | --- | --- | --- |
| SOD (MA1574) | 5´CCCTTCTGGAAATGATGGAC3´ | 5´TTGCTTGCAGGAGTCATCTC3´ | 139 |
| CAT (MA0972) | 5´CGGAGCAAACAAACAAGAAA3´ | 5´TGAACTCCTTCGCGTAATTG3´ | 132 |
| PX (MA1426) | 5´AAAGCAGCCGTAAATGTTCC3´ | 5´GAAGTCAGGTTCGCTGATGA3´ | 132 |
| Reference gene (MA3998) | 5´TGATCGAGAAACTGGCAGAC3´ | 5´TGCCTGACCATGGATACACT3´ | 115 |
